# Evolutionary Overview of Terrace Research Based on Bibliometric Analysis in Web of Science from 1991 to 2020

**DOI:** 10.3390/ijerph19137796

**Published:** 2022-06-25

**Authors:** Qianru Chen, Yuyang Wen, Xinmin Zhang, Zhenhong Zhu

**Affiliations:** Institute of Ecological Civilization, Jiangxi University of Finance and Economics, Nanchang 330013, China; 2201920708@stu.jxufe.edu.cn (Y.W.); 1201800118@jxufe.edu.cn (X.Z.); 2202010105@stu.jxufe.edu.cn (Z.Z.)

**Keywords:** agricultural terraces, land use, bibliometrics, co-word analysis, thematic evolution

## Abstract

Based on the Web of Science core collection database, this paper retrieves 349 research papers on terraced fields published during 1991–2020. Keyword co-occurrence analysis, cluster analysis, and thematic evolutionary analysis were used to identify the evolutionary path of terrace research. The findings were as follows: (1) In the past 20 years, the study of terraced fields has shown an upward trend. The number of annual published papers during 2012–2020 was much more than that during 1991–2011, but papers during 1991–2011 were more academically influential than those during 2012–2020. (2) Regional analysis showed that terrace research in China is the most abundant currently, and is mainly focused on agricultural production, agricultural engineering, cultural tourism, and ecological environment. (3) Keyword co-occurrence analysis showed that terrace landscape, terrace agriculture, terrace abandonment, land use change, soil and water conservation, and sustainable utilization of typical terraces are the main modules of current terrace studies. (4) In a temporal dynamic perspective, terrace research presented 10 main evolutionary paths during 1991–2020, reflecting the trend of terrace research towards sustainable terrace development of ecological agriculture and ecosystem service. (5) Finally, this paper suggests that here is a need to deepen studies on terrace ecosystem services and human well-being based on their structure and processes, to analyze the interaction and comprehensive effect of natural process and humanistic driving forces on terrace abandonment, and to explore the multi-functional benefits and sustainable management of high quality terraced landscape.

## 1. Introduction

Terraces refer to cultivated land with terraced distributions, horizontal surfaces, and ridges [1]. Terraced fields are recognized as living fossils created by human beings in nature, and they are great symbols of human utilization and transformation of nature. Terraces built at different times are scattered all over the world, especially in Asia and Europe. Terraced fields first appeared in prehistoric times. Terraced fields were initially used to build fortifications on hillsides and to cultivate land. Later, they evolved into an intensive agricultural production mode on mountains all over the world [2]. Nowadays, various new terraces have been built in mountainous and hilly areas, and they can be divided into different types according to different classification standards. For example, terraces can be divided into table terraces and wave terraces by their cross-section structure [3]. They can be divided into horizontal terraces, slope terraces, reverse slope terraces, and separated slope terraces by the field surface structure, among which the horizontal terrace is the most common [4]. Terraces can be divided into earth terraces and stone terraces by construction material. In modern times, cement terraces have become more and more common due to their role in landslide prevention and control. In addition, terraces can be divided into rice terraces and dry farming terraces, where cash crops such as grapes and kiwifruit are usually planted. According to literature review, current research on terraces mainly focuses on their history and distribution, and their value and utilization. The world-famous terraces listed on the World Cultural Heritage list by UNESCO, including the Honghe Hani rice terraces and Yuanyang terraces in China, the Ifugao rice terraces in the Philippines, and the Lavaux vineyard terraces in Switzerland, have attracted most scholars’ attention in fields such as agricultural planting benefits, soil and water conservation, and landscape aesthetics.

Terraced fields are of great value in broadening agricultural production sources, alleviating soil erosion and degradation [5,6], saving agricultural water resources, reducing runoff, improving biodiversity [7], and increasing ecological and cultural values. Terrace archaeology has also shown that terraced fields in different periods were built for different reasons, such as increasing per capita cultivated land area and grain output, soil and water conservation, and cultural landscape promotion. Due to the limitations of population and terrain, people in areas with low per capita cultivated land area have to develop terraces in hilly areas to increase food output. Modern scientific research in areas with severe soil erosion has proven that terraced fields effectively slow surface runoff [8,9,10]. Terraced fields help reduce water loss in soil [11] and prevent the deposition of soil organic matter from terraces on the bottom of sloping land or prevent losses [12,13]. Some studies have also found that terraced fields play an important role in microclimate and flood relief [14,15]. More than material practicability, the value of terraced fields is also reflected in intangible cultural value [16], such as providing landscape aesthetics [17,18,19] or cultural heritage [20,21].

Although terraced fields are of great value, their utilization is not optimal. Studies show that influenced by agricultural input cost, grain yield, agricultural facilities, labor quantity, and labor opportunity cost [22,23,24,25,26], the abandoned terrace area has reached 77.4% in the past 60 years [27]. Compared with ordinary farmland, terraced fields are more likely to be abandoned due to poor farming, irrigation, or transportation conditions, which will result in low grain yield. Especially in recent years, with the rapid development of secondary and tertiary industries, the proportion of farmers’ agricultural income continues to decrease, while the cost of farming continues to rise. In this context, terraced fields will be abandoned preferentially among farmers’ contracted land.

The influence of terraced land abandonment is multifaceted. Although terrace abandonment may benefit biodiversity improvement and soil habitat restoration, its adverse effects cannot be ignored. Research show that terrace abandonment will cause resource waste, ecosystem service value reduction, land cover change, and an overall decline in water resource availability [28], resulting in wildfire risk and land degradation [29,30]. However, the adverse influence of abandonment will diminish over time [31].

More than typical ecological agriculture modes in mountainous areas, terraced fields are also outstanding ecological and cultural landscapes and precious agricultural cultural heritage, which have important enlightenment and reference significance to the sustainable development of agriculture. The second section of this paper introduces data sources and research methods. The third and fourth sections describe quantitative and qualitative scientometric analysis on terraced fields by bibliometrix and VOSviewer software, respectively. The fifth section draws conclusions and describes prospects for future research. This review provides a broader vision of terrace study, which has a positive effect on terrace landscape maintenance, ecological protection, and sustainable development in hilly and mountainous areas.

## 2. Data Sources and Research Methods

### 2.1. Data Sources

The data in this study were sourced from the world’s largest comprehensive information resource, the Web of Science core collection database of the Institute for Science Information (ISI). This database includes over 8700 core academic journals in various fields of natural sciences, engineering, biomedicine, social sciences, arts, and humanities. In this paper, the core collection of the Web of Science was used as the data source, the SCI-EXPANDED and SSCI databases were indexed, articles were selected as the literature type, and English was set as the language. After screening and removing the duplicated data, 349 papers on terraced fields during 1991–2020 were obtained, and the downloaded data was saved in a text format.

### 2.2. Research Methods

**Figure 1:**
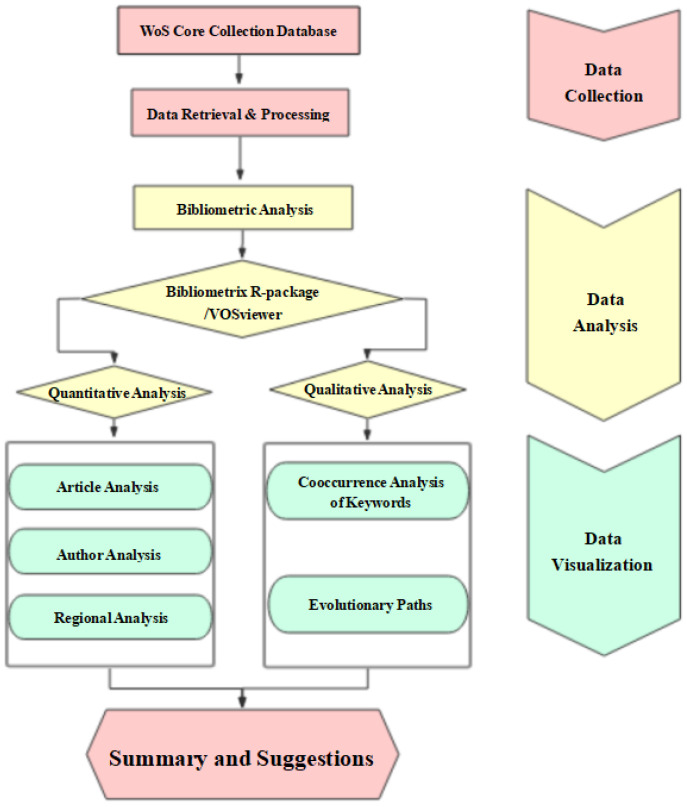
Bibliometrix and science-mapping workflow.

Bibliometric analysis provides a comprehensive overview of a large body of research literature and further develops previously unevaluated insights by allowing quantitative and objective identification of past and present research topics [32]. The Bibliometrix *R* package provides a set of tools for quantitative research in scientific metrology. R-language-based bibliometric software developed by Dr. Aria and others provides support for importing and processing literature information from SCOPUS and WoS, statistically analyzing relevant scientific literature indices, constructing a data matrix, and conducting research and visualization processing on co-citation, coupling, cooperative analysis, and co-word analysis [33]. It has been widely used in scientific research in various fields in recent years [34,35,36]. VOS viewer, developed by Jan van Eck and Ludo Waltman, is widely used in different kinds of co-occurrence analysis software, especially in keyword co-occurrence analysis [37,38,39,40,41]. To accurately and comprehensively analyze the research status and popular topics on terraced fields, this study used VOS viewer and bibliometrix software packages to analyze and visualize current research on terraced fields. This paper analyzes the annual number of published papers, research power (country and author), and popular research topics quantitatively and qualitatively. At the same time, this paper analyzes the trends in terrace research through historical citation analysis, theme evolution analysis, and coupling analysis. The specific research steps are shown in Figure 1.

## 3. Summary of Quantitative Research

### 3.1. Article Publication and Citation Analysis

Based on the statistical analysis of 349 papers on terraces during 1991–2020 from WoS, the research was divided into two stages. The first stage was the exploration of the period of low yield from 1991 to 2011, which was characterized by the small number of published papers on terraces. In this period, the average number of annual published papers was 7.1 and the average annual number of citations was 23.1. The second stage was the period of increasing production during 2012–2020. The average number of annual published papers had increased to 21.7 and the average annual citations reached 10.9. While the number of published papers in the second stage was approximately three times that of the first stage, the number of citations in the second stage was only half that of the first stage, indicating a greater academic influence in the first stage and a higher research enthusiasm in the second stage. It also indicates that the early pioneering studies from varied perspectives laid a foundation for subsequent terrace research. Based on current research trend, it is expected that terrace research will be further enriched.

**Figure 2:**
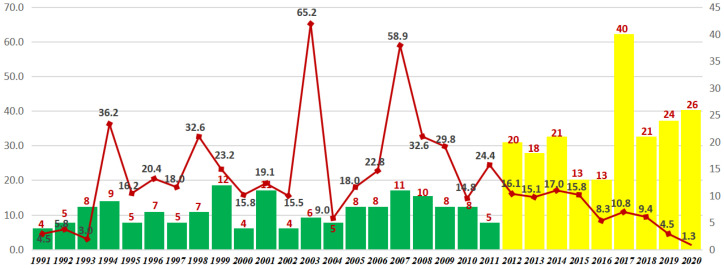
Agricultural-terrace research articles and citations during 1991–2020.

In the first stage during 1991–2011, there were four small peaks of citations, namely, 1994, 1998, 2003, and 2007 (line chart of average citations in Figure 2). The paper “*Studying the role of old agricultural terraces on runoff generation in small Mediterranean mountainous basin*” published in 1994 had been cited 122 times by the end of the study period. It was proven in this paper that surface runoff in the Mediterranean mountainous area was related to the spontaneous reorganization of the artificial drainage network in abandoned terraced fields [42]. By the end of 2020, the article “*Land use change effects on abandoned terraced soils in a Mediterranean catchment, NE Spain*” published in 2003 had been cited 146 times. In this paper, the different vegetation stages were divided into four main land-use types according to the age of abandonment: cultivated fields (vineyard and olive tree, 0 years), recent abandonment (dense and cleared shrubs, 5 years), moderate abandonment (cleared cork trees and dense olive trees, 25 years) and early abandonment (dense cork trees and pine trees reforestation, 50 years), and variance analysis indicated significant differences in the main soil quality parameters such as soil organic matter (SOM), total nitrogen (N), water holding capacity (WHC), and pH, among the selected environments under different land-use conditions [43]. The paper “*Land environment and slope gradient as key factors of soil erosion in Mediterranean terraced lands*” published in 2007 had been cited 211 times by the end of 2020. The research results showed that the influence of traditional extensive cultivation abandonment on soil sediment losses varied with slope gradient. When slope gradient was steep (25%), soil erosion increased significantly. When slope gradient was very steep (40%), soil sediment losses remained at the same high levels after cultivation abandonment as slope gradient was the main factor controlling soil erosion, although soil and vegetation properties were changing [44]. The highly cited papers in the first period solved key basic problems such as classification standards and environmental impacts in the initial stage of terrace research through exploratory attempts, so their influence was greater. The average number of annual published papers and citations are shown in Figure 2 below.

### 3.2. Analysis of Authors’ Papers

**Figure 3:**
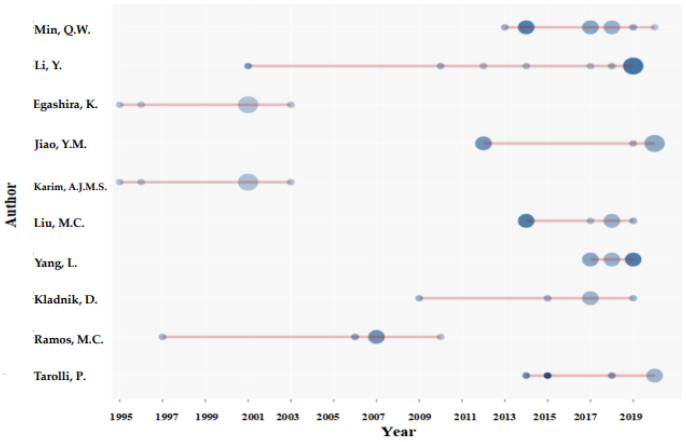
Authors’ publications on agricultural terraced fields over time.

As shown in Figure 3, the bubble chart of authors’ papers directly reflects the published number of articles and citations of each author in different years. The circle size represents authors’ published article numbers, the color depth represents the number of the paper’s citations. The top ten authors in terrace research field are shown in Figure 3. H-indexes, total numbers of published articles, total citations, and publication dates of the ten authors are shown in Table 1. An author’s H-index means that at least H of his/her papers had been cited at least H times in a given period [45]. It can be seen from Table 1 that the H-index of Min Q. W. and Li Y. was 5, indicating that their studies were the most influential. Taking rice-fish system in Hani Terrace as an example, Min Q.W. studied the standards of ecological compensation for traditional eco-agriculture [46]. Li Y. studied crops planted in terraced fields from a biological viewpoint for 19 years. He proposed that the accumulation of H_2_O_2_ and MDA helped induce the improvement of antioxidant enzyme activity, thus improving the tolerance of plants to UV-B radiation. The enhancement of UV-B radiation directly affected rice growth, and changed the system of *Magnaporthe grisea* indirectly, which was conducive to terraced-field planting [47,48]. Li Y.’s team quantified the soil quality parameters of terraced and steep slopes on the Loess Plateau of China, and cleared the influence of tillage erosion and water erosion on soil quality parameters. It was concluded that water erosion was the main reason for the overall decline in the soil quality of steeply sloping farmland, and that tillage had a controlling effect on the spatial pattern of organic matter, nitrogen, and phosphorus in terraced and steep-slope soil [49]. These studies on terraced fields from different angles, such as economy, ecology, and landscape, have laid a foundation for multidisciplinary comprehensive study of terraced fields.

### 3.3. Analysis of National Documents

#### 3.3.1. Global Research Analysis

**Figure 4:**
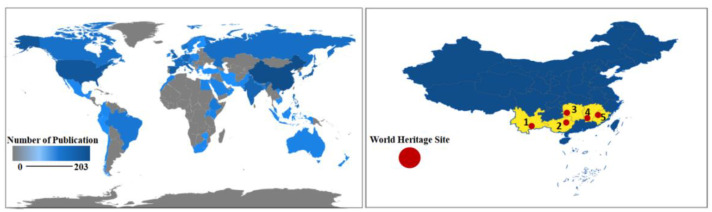
Number of publications on terraced fields in each region of the world.

In Figure 4, the shades of blue indicate the number of papers published in the country, the gray indicates that there is no relevant literature on terraces in the country, the red dots indicate the location of the 20 countries with the largest number of papers, and the yellow triangle indicates the famous terraces that have been listed as world heritage sites by FAO or UNESCO. The distribution map of numbers of papers published by different countries shows that there is little research on terraces in Africa and Western Asia. As can be seen from Table 2, related articles published in China are more abundant, indicating a strong research force and phased research focus in China. However, in terms of research influence, articles from developed countries in Europe and America seem to be more influential, which can be seen from the higher citations of articles from developed countries. On the whole, research on terraces is consistent with the distribution of terraced fields, that is, terrace research is more abundant in regions with more terraced fields, such as East Asia, Western Europe, and North America. Asian terraces are mainly planted with rice, and these large-scale terraced landscapes support a large population and form a common and distinct “terraced cultural circle” [50], while European terraces were mainly built to facilitate the mechanized production of vineyards during their long history. They are represented by the Lavaux vineyard terraces in Switzerland, and terraces in Hungary, Italy, Portugal, and Spain. However, agricultural industrialization and abandonment in Europe led to the degradation and disappearance of traditional terraced landscapes after the 1960s.

#### 3.3.2. Analysis of Major Research Countries

China is the country with the largest number of publications. There are 203 articles written by Chinese authors, accounting for 58.2% of the total publications. Due to the large population and limited per capita cultivated land area in China, the Chinese government attaches great attention to terrace development and protection to ensure food security. In recent years, owing to the Transforming Slope into Terrace Project on the Loess Plateau, terraced area has increased significantly in China [51,52,53,54]. Data from WoS show that the five Chinese world terraced fields, namely Honghe Hani Terraced Fields, Ziquejie Terrace, Longji Terrace, Youxi United Terrace, and Chongyi Hakka Terraces, are the main research areas of Chinese publications (Table 3, Figure 4).

**Figure 5:**
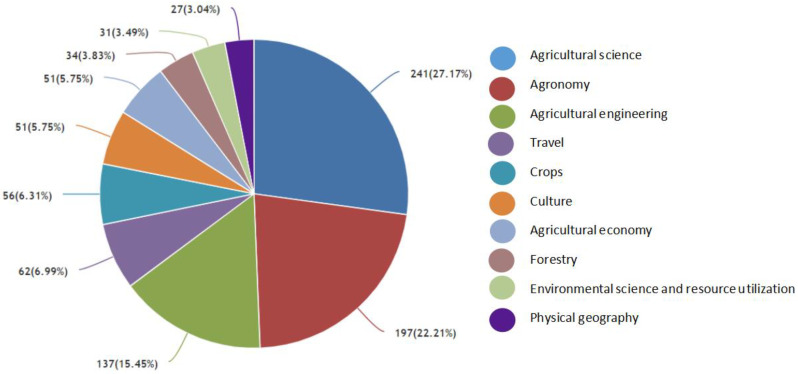
The main terrace research subjects in China.

Discipline analysis shows that terrace research in China is mainly focused on the following four aspects (Figure 5). ① Agricultural production. Rice and local rice varieties are the main varieties, among which, red rice in Hani Terrace is a traditional high-quality variety. ② Agricultural engineering. To solve soil and water loss in the Loess Plateau and control Yellow River bed aggradation, some scholars suggest transforming sloping land into terraced farmland, so as to reduce nutrient loss on the surface of cultivated land. In addition, the identification and extraction of terrace spatial information has developed rapidly in recent years, but its application on a large scale still needs to be improved. ③ Cultural tourism. Represented by Hani Terrace and Longji Terrace, terraced field tourism combines visual enjoyment brought by terraced landscapes and ethnic culture, thus bringing positive travel opportunities and economic benefits. In this way, a new terrace-tourism mode combining terrace protection with landscape, local culture, and tourism revenue, can be formed [55,56,57]. ④ Ecological environment. Biodiversity conservation, ecological compensation, and ecosystem service value, especially terraced cultural service value which involves cultural, aesthetic, spiritual, and religious aspects, has attracted more and more attention in terrace field [58,59].

## 4. Summary of Qualitative Research

### 4.1. Analysis of Keyword Co-Occurrence Network

**Figure 6:**
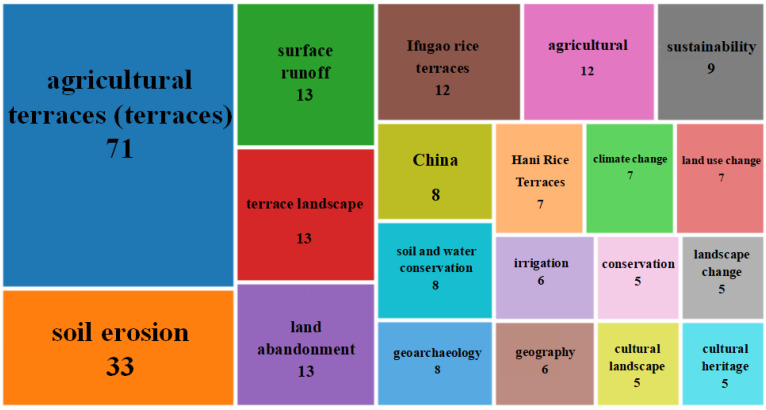
High frequency keywords and their occurrence in agricultural terrace publications.

**Figure 7:**
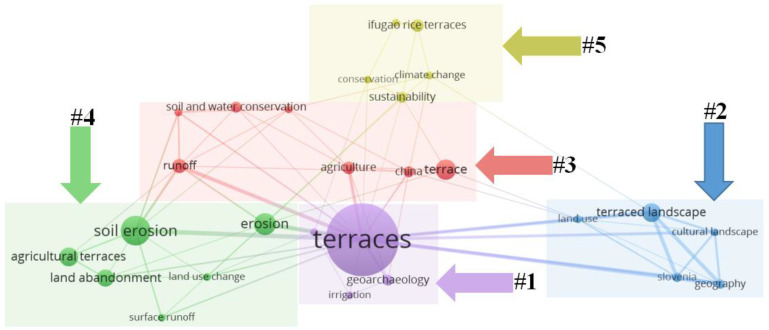
Co-occurrence network of agricultural terrace keywords.

The 20 most frequent keywords in terrace studies were obtained by eliminating meaningless or repetitive words through statistics, as shown in Figure 6. “Agricultural terraces” was the most frequent keyword, followed by “soil erosion”, “surface runoff”, “terraced landscape”, and “land environment”. A single keyword can identify the core information of terrace research, but it cannot identify the necessary relationship between the core information. Therefore, the co-occurrence analysis in VOS viewer was introduced to set the author keywords at least five times to obtain keyword co-occurrence (Figure 7). In Figure 7, the lines represent the usage of keywords. The larger the node, the higher the frequency of keywords. The five main clusters of keywords, labeled #1–#5, in terrace studies are highlighted by rectangular boxes of different colors in Figure 7. They also reflect the main modules of current terrace studies.

Cluster 1 mainly focuses on the geological and archaeological characteristics of terraced fields. Geoarchaeology not only helps understand terrace history, function, and sustainability, but also provides evidence for vegetation change and erosion over time [60]. Terrace construction is a long process. Terraces are rarely constructed in a single stage but gradually develop or even evolve, and new terraces are usually built gradually based on maintaining ancient terraces [2,61,62]. Archaeological study of isotopic carbon or nitrogen on terraced soil indicates that the Longji terrace in China was constructed in the late Chinese Yuan Dynasty. In the late Yuan Dynasty, the strong survival pressure brought by social upheaval motivated minority populations to migrate to the Longji Mountain area to open up new living space. As a result, agricultural terraces and gravity irrigation networks were built on hillside land [63]. Archaeological research in Bali proved that the spatial pattern of Balinese terraces built hundreds of years ago was more about farmers’ decision-making and rice field ecology [64].

Cluster 2 mainly involves studies on terrace landscape and its cultural value. Cultural landscape is formed in the long-term interaction between humans and nature [65]. It also represents humans’ living landscape formed by long-term intensive and continuous cultivation [66]. As the cultural value of terraced landscape is derived from the combination of terrace maintenance and value creation [67], Japan attaches great importance to the protection and inheritance of terraced landscape culture. Local people believe that terraced field is the manifestation of hard work, which represents people’s belief in natural spirit [68]. In addition, beautiful terrace landscape brings visual enjoyment to tourists and even affects people’s world outlook, which tourists are willing to pay for. In many places, owing to their cultural value, terraces are mainly retained as decorative elements of the landscape rather than for their original agricultural function although farmers actively cultivate their fields [69]. Especially in some tourist attractions, terrace cultivation is maintained mainly to protect its cultural landscape value, which is much higher than its food production value.

Cluster 3 mainly involves studies on soil and water conservation and agricultural utilization of terraced fields. Traditional slope tillage causes severe soil erosion, while terrace construction helps prevent soil erosion effectively [70]. However, the effectiveness is limited by factors including climate, soil properties, topography, land use, culture, population, and socioeconomic status [71]. To maximize the function of terraces in soil and water conservation, numerous studies have been carried out. It is proposed that terraces should be well maintained to retain more water, and vegetation restoration is essential in areas prone to water flow [72,73]. Studies in abandoned terraced fields, which are more prone to soil erosion, show that terraces planted with vegetation produce less runoff than those planted with crops [74]. Therefore, adjusting planting types and avoiding terrace abandonment helps reduce soil erosion.

Cluster 4 mainly involves studies on terrace soil erosion, land abandonment, and land-use change. In recent years, with the development of the social economy, farmland abandonment has become more and more serious [75]. In comparison to flat land, abandonment is more likely to occur in terraced fields due to their inferior topography and irrigation conditions. Currently, studies on terrace abandonment are mainly focused on the identification [76], driving forces, impacts, and recovery. Some reasons for cultivated land abandonment and terrace abandonment might be similar, but the degree of influence is different. For example, the influence of slope on abandonment is greater for terraces than for cultivated land [44]. Terrace abandonment may cause soil erosion and wildfires [30], and further reduce biodiversity and landscape cultural value [77]. It is noteworthy that soil erosion and terrace abandonment are mutually causal. Therefore, to alleviate the adverse effects of terrace abandonment, it is proposed to restore and maintain terraced fields. Local endemic species are encouraged to be replanted to promote the restoration of local ecosystems, so as to enable the ecology and land use in terraced fields to evolve in a better way [78].

Cluster 5 mainly involves studies on the protection and sustainable utilization of typical terraces, which are represented by the Ifugao rice terraces. Although some ancient terraced fields function fully, it cannot be ignored that inefficient utilization still widely exists. Combining historical and cultural landscape perspectives, scholars have studied well-maintained and typical terraced fields to seek the sustainable utilization of terraced fields. Studies have found that both natural and social-economic factors affect the sustainable use of terraced fields. For example, the El Niño phenomenon has caused drought and insufficient irrigation infrastructure in the Ifugao rice terraces, resulting in decreased ecosystem value, and obvious changes in extensive planting and ripening [79]. At the same time, the influence of organisms is striking too. In the Ifugao rice terraces, golden apple snails seriously harm crops, and earthworms and mice disintegrate terraces [80,81]. Similar problems exist in the Hani terraces. The introduction of new rice varieties significantly increases the plant diseases and insect pests [82]. In addition, extensive management and abandonment begin to spread as farmers’ willingness to plant decreases. To realize the sustainable utilization of terraced fields, dynamic protection approaches are proposed. First, it is necessary to enhance farmers’ cultural cognitive abilities, especially their consciousness to traditional agricultural knowledge. Second, it is necessary encourage young people to participate in the management and maintenance of terraced fields [83,84]. Third, there is a need to improve the ecological compensation mechanism to promote ecological agriculture and ecological services [85]. For terraced areas with cultural value, tourism development and community monitoring can be combined to explore an endogenous development strategy [86].

The above analysis shows that the five clusters are closely related and mutually supported. Despite their own focus, the contents of the five clusters are both dependent and intersecting, reflecting the interdisciplinary development trend in terrace research. As a whole, the five clusters reflect the general research situation of terraced fields involving utilization, functions, values, and development.

### 4.2. Analysis of Thematic Evolution

**Figure 8:**
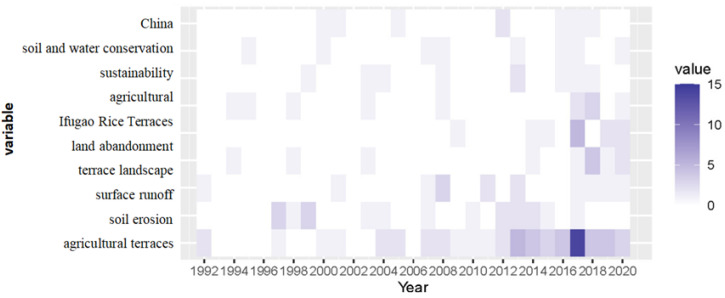
Thermal map of terrace research.

**Figure 9:**
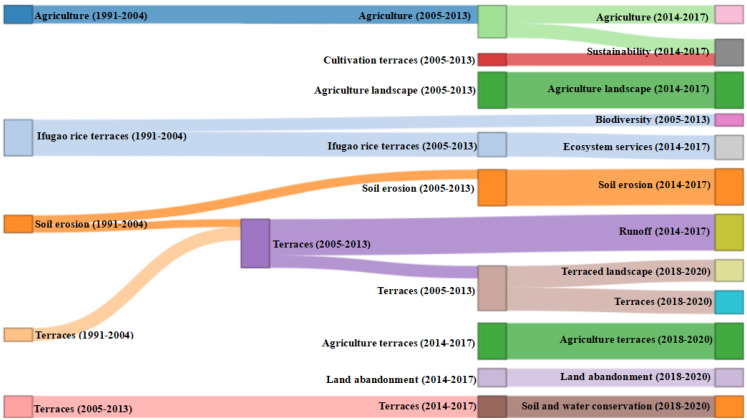
Thematic evolution of agricultural-terrace research (1991–2020).

To further analyze the research context reflected by keywords, Figure 8 is charted to present the temporal information of keywords and research hotspots, thus providing support to study the relationship between different research fields from temporal and causal dimensions. In Figure 8, the darker the color, the hotter the research. To visualize the theme evolution of terrace research over time, a Sankey diagram (Figure 9) is charted to present the qualitative information and flow status of different themes [87], such as theme flow and its direction and transformation relationships.

Three evolutionary time nodes of 2004, 2013, and 2017 were given through the software. Figure 9 shows that both single research paths and extended research paths exist in terrace research over time. The single research path means that the research theme remained unchanged during the study period, and continues to be the research focus for a period of time in the future. Single research paths are divided into traditional paths and emerging paths based on when they formed, among which, emerging paths are more likely to become popular. For extended research paths, the former and latter research themes are closed related but different; to be specific, the former enlightens the latter while the latter deepens the former. 

The four main single research paths are as follows:

(1) Agriculture (1991–2017);
(2) Soil erosion (1991–2017);
(3) Agricultural landscape (2005–2017);
(4) Land abandonment (2014–2020).

According to the study period, paths 1, 2, and 3 are traditional paths, while path 4 is an emerging path. The temporal information shows that early terrace research (1991–2017) focused on agricultural and soil erosion. Traditional terraces were generally built on hillsides to develop cultivated land, regulate water circulation, and maintain soil. The early research paths were consistent with the original development needs for terraced fields. Namely, to control soil and water loss of production land by slope treatment, and to improve soil physical and chemical properties through terrace cultivation to accumulate nutrients needed for crop growth, thus improving agricultural production capacity. Terraced fields play an important role in maintaining slope landscape pattern and tourism development by changing land landscape and increasing regional landscape heterogeneity. Therefore, with the development and improvement of terrace engineering technology, scholars pay more attention to the agricultural landscape value of terraced fields, including landscape pattern change, typical watershed landscape patterns, terraced cultural landscapes, comprehensive evaluation of landscape multifunctional value, and landscape protection. In recent years, due to the decline of agricultural comparative income, the rise of agricultural opportunity cost, and the constraints in terrace engineering design, abandoned terrace fields gradually expanded and became an emerging research focus. The advancement of industrialization and urbanization highlights the intensifying terrace abandonment. Therefore, study of abandoned terraced fields will continue to be a hotspot in future.

The expanded research paths are as follows:

(1) Cultivation terraces (2005–2013) → sustainability (2014–2017);
(2) Ifugao rice terraces (1991–2004) → biodiversity (2005–2013);
(3) Ifugao rice terraces (1991–2013) → ecosystem services (2014–2017);
(4) Soil erosion (1991~2004) → terraces (2005–2013) → runoff (2014–2017);
(5) Soil erosion (1991~2004) → terraces (2005–2017) → terraces landscape (2018–2020);
(6) Terraces (2005~2017) → soil and water conservation (2018–2020).

The evolutionary process of expanded research paths is more abundant than the single research paths. Path (1) reflects the sustainability goals and research trends of terrace cultivation, including the necessity of terrace sustainable development [88], sustainable terrace management [89], and sustainable terrace practice mode and its evaluation [90,91]. For example, Ni (2014) believed that the Hani terrace in China should develop small-scale sustainable and ecological agriculture, and take this as an opportunity to develop ecological agricultural tourism [92]. Paths (2) and (3) reflect the importance of the studies on the ecosystem service value of rice terraces. Among them, the literature on the Ifugao rice terrace in the Philippines is the most abundant and representative. For example, the study on the resilience of the Ifugao terraced agricultural system provides a reference for evaluating the biodiversity and sustainable development of terraced ecosystems. More than food supply, the functions of a rice terrace ecosystem with rice as the main crop also include climate regulation [93], soil and water conservation [94], pest regulation, tourism [95,96], and aesthetics [97]. The cultivation and development of rice terrace is of great significance in maintaining ecological security and food security in mountainous areas. Biodiversity maintenance is a research hotspot of terrace ecosystem service. For example, Drechsler and Settele (2001) studied the predator–prey interactions in rice ecosystems of Philippine rice terraces, thus exploring the effects of guild composition, trophic relationships, and land use changes on terrace biodiversity [98]. These studies provide important support for the realization of ecosystem services. Paths (4), (5), and (6) are similar in research content regarding the effective management of water and soil resources in terraced fields, including terrace soil erosion and runoff and its countermeasures. Currently, soil and water conservation measures have also become an important part of the terraced landscape, that is conducive to the planning and stability of landscape pattern. As the role of soil and water conservation in maintaining terrace landscape ecology highlights, the theme of path (5) develops from soil erosion (1991–2004) to terrace landscapes (2018–2020).

The theme evolution pathways present a terrace research trend from single to diversified and from simple to complex in the past 30 years. Themes have evolved from the initial topics of agriculture, terraces, and soil erosion to sustainable utilization, biodiversity, and ecosystem services. However, research on the classified management of terrace fields is still deficient, and research on terrace historical evolution, value evaluation, eco-environmental effect analysis, and specific management strategies is expected to become popular in the future.

## 5. Conclusions and Suggestions

This paper conducted quantitative and qualitative analysis on literature of terraced fields from 1991–2020 from the WoS database. The quantitative analysis included article, author, and regional analysis, and the main conclusions were as follows: (1) terrace research presents an increasing trend with fluctuations during 1991–2020; (2) terrace research has attracted scholars from various fields, and is expected to remain a hotspot in the future; and (3) agricultural production, agricultural engineering, cultural tourism, and ecological environment are the main research aspects in China. Qualitative studies included keyword co-occurrence and thematic evolution analysis. The main conclusions were as follows: (1) keyword co-occurrence analysis formed five research clusters focused on terrace geological archaeology, terrace landscape and cultural value, the effectiveness of terrace soil and water conservation, and the utilization and change of terraces, reflecting the main modules of current terrace studies and (2) thematic evolution analysis presented 10 main evolutionary paths of terrace research in a temporal dynamic perspective, which were characterized by single and expanded research paths and included “land abandonment (2014–2020)” and “soil erosion (1991–2004) → terraces (2005–2017) → terraces landscape (2018–2020)”, reflecting the trend of terrace research towards sustainable terrace development of ecological agriculture and ecosystem service. By reflecting terrace research emphases in different periods, thematic evolution analysis provides ideas for analyzing the evolutionary process and mechanism of terraced field research, thus providing a basis for understanding the future research direction of terraced fields. Based on the existing literature, this paper puts forward the key content and direction of terrace research in future.

(1) As important agricultural landscapes and cultural heritages, terraced fields have become a model of the coupling between man and nature, and also a unique way for humans to adapt to the mountain ecosystem during their long-term struggle against harsh natural conditions. Terrace ecosystems provide a variety of services for mankind, and their ecosystem structure and processes affect the service supply. However, there is little research on the spatial–temporal matching of terrace ecosystem services in current studies, thus failing to reveal the interaction between multiple services from the service formation mechanism, and research on the evaluation of terrace ecosystem services is also lacking. Therefore, based on the structure and processes of terrace ecosystems, future studies may focus on terrace ecosystem services and human well-being, and carry out quantitative and qualitative evaluation of terrace ecosystem services according to the research environment and purpose.

(2) Terrace research shows a comprehensive and diversified trend in terms of research content, research scope, and research scale. However, studies on the driving forces and risk assessment of terrace abandonment are few. In view of the complexity and coupling effect of terrace abandonment, it is necessary to give full play to the multi-disciplinary advantages of natural ecology, sociology, economics, geography, and human policies and to take into account the demands of stakeholders including scientists, decision makers, grass-roots managers, and farmers, thus comprehensively analyzing the interaction and effect of natural process and humanistic driving forces on terrace abandonment.

(3) There is a need to study countermeasures to strengthen ecological protection and restoration of terraced fields, and to explore the multi-functional benefits and sustainable management of high-quality terraced landscapes. In fact, under the impact of the modern scientific and technological revolution and the new ways of production and life, terrace ecosystems in most parts of the world are threatened by abandonment and extinction. It is necessary to establish a long-term self-sustaining mechanism of terrace ecosystems through dynamic protection and adaptive management of terrace landscapes, so as to realize the sustainable utilization and development of terraced multi-functional landscapes.

## Figures and Tables

**Table 1:** Top 10 authors in the field of agricultural-terrace research.

Author	H_Index	TC	NP	PY_Start
Min Q.W.	5	96	9	2013
Li Y.	5	121	7	2001
Egashira K.	3	20	6	1995
Jiao Y.M.	4	53	6	2012
Karim A.J.M.S.	3	20	6	1995
Liu M.C.	4	69	6	2014
Yang L.	4	41	6	2017
Kladnik D.	4	49	5	2009
Ramos M.C.	4	159	5	1997
Tarolli P.	3	137	5	2014

Notes: TC: total article citations; NP: number of papers; PY_start: publication year.

**Table 2:** Top 10 countries in terms of article number.

Region	Articles	Total Citations	Average Number of Article Citations
China	203	732	3.61
USA	95	784	8.25
Japan	62	224	3.61
Spain	60	1136	18.93
Italy	38	401	10.55
United Kingdom	35	510	14.57
India	30	40	1.33
Germany	28	234	8.36
Philippines	26	85	3.27
France	18	74	4.11

**Table 3:** Main terraces in China.

Name	Location	Area (hm)	Crop	World Heritage Site
Honghe Hani Terraced Fields	Yunnan	16,603	Rice	UNESCO(2013), GIAHS(2010)
Longji Terrace	Guangxi	7010	Rice	GIAHS(2018)
Ziquejie Terrace	Hunan	1334	Rice	GIAHS(2018)
Chongyi Hakka Terraces	Jiangxi	2000	Rice	GIAHS(2018)
Youxi United Terrace	Fujian	713	Rice	GIAHS(2018)

## Data Availability

Not applicable.
